# p38β MAPK upregulates atrogin1/MAFbx by specific phosphorylation of C/EBPβ

**DOI:** 10.1186/2044-5040-2-20

**Published:** 2012-10-09

**Authors:** Guohua Zhang, Yi-Ping Li

**Affiliations:** 1 Department of Integrative Biology and Pharmacology, University of Texas Health Science Center at Houston, 6431 Fannin Street, Houston, TX, 77030, USA

**Keywords:** Cachexia, E3 protein, Gene regulation, DNA-binding, Thr-188

## Abstract

**Background:**

The p38 mitogen-activated protein kinases (MAPK) family plays pivotal roles in skeletal muscle metabolism. Recent evidence revealed that p38α and p38β exert paradoxical effects on muscle protein homeostasis. However, it is unknown why p38β, but not p38α, is capable of mediating muscle catabolism via selective activation of the C/EBPβ that upregulates atrogin1/MAFbx.

**Methods:**

Tryptic phosphopeptide mapping was carried out to identify p38α- and p38β-mediated phosphorylation sites in C/EBPβ. Chromosome immunoprecipitation (ChIP) assay was used to evaluate p38α and p38β effect on C/EBPβ binding to the atrogin1/MAFbx promoter. Overexpression or siRNA-mediated gene knockdown of p38α and p38β, and site-directed mutagenesis or knockout of C/EBPβ, were used to analyze the roles of these kinases in muscle catabolism in C2C12 myotubes and mice.

**Results:**

Cellular expression of constitutively active p38α or p38β resulted in phosphorylation of C/EBPβ at multiple serine and threonine residues; however, only p38β phosphorylated Thr-188, which had been known to be critical to the DNA-binding activity of C/EBPβ. Only p38β, but not p38α, activated C/EBPβ-binding to the atrogin1/MAFbx promoter. A C/EBPβ mutant in which Thr-188 was replaced by alanine acted as a dominant-negative inhibitor of atrogin1/MAFbx upregulation induced by either p38β or Lewis lung carcinoma (LLC) cell-conditioned medium (LCM). In addition, knockdown of p38β specifically inhibited C/EBPβ activation and atrogin1/MAFbx upregulation induced by LCM. Finally, expression of active p38β in mouse tibialis anterior specifically induced C/EBPβ phosphorylation at Thr-188, atrogin1/MAFbx upregulation and muscle mass loss, which were blocked in C/EBPβ-null mice.

**Conclusions:**

The α and β isoforms of p38 MAPK are capable of recognizing distinct phosphorylation sites in a substrate. The unique capacity of p38β in mediating muscle catabolism is due to its capability in phosphorylating Thr-188 of C/EBPβ.

## Background

The p38 mitogen-activated protein kinases (MAPK) family plays a pivotal role in skeletal muscle by mediating diverse cellular activities, and interestingly, some of which result in paradoxical effects. For example, p38 mediates both insulin stimulation of glucose uptake [1] and TNF-α stimulation of insulin resistance [2] in muscle. In the context of skeletal muscle protein homeostasis, p38 responds to both catabolic (lipopolysaccharide, cytokines, and ROS) [3-5] and anabolic (insulin and exercise) stimuli [6]. On one hand, p38 stimulates muscle satellite cell proliferation [7] and differentiation [8], which increases muscle mass; on the other hand, p38 stimulates muscle protein degradation leading to muscle atrophy [3-5]. Intriguingly, p38 has the capacity to activate different protein substrates depending on the cellular environment [7]. It is of great interest to understand how the p38 MAPK family is able to mediate the discrete and sometimes opposing effects in response to diverse physiological and pathological stimuli. The family of MAPK is composed of at least four members (α, β, γ and δ), which enable the transduction of a variety of extracellular signals into distinct nuclear responses [9-11]. The α, β, and γ isoforms are found in muscle cells. Recently, it became clear that p38α MAPK plays an essential role in myogenic differentiation [12,13]. On the other hand, p38γ MAPK appears to regulate the expansion of myogenic precursor cells [14], endurance exercise-induced mitochondrial biogenesis and angiogenesis [15], as well as glucose uptake [16]. But, little was known about the function of p38β until our most recent discovery of its role in regulating the atrogin1/MAFbx gene [17].

Cachexia, a wasting disease characterized by loss of muscle mass with or without loss of fat mass, is frequently associated with such diseases as cancer, sepsis, AIDS, congestive heart failure, diabetes, chronic renal failure and chronic obstructive pulmonary disease (COPD). Cachexia is distinct from starvation-, disuse-, aging-, primary depression-, malabsorption- and hyperthyroidism-induced muscle mass loss and is associated with increased morbidity and mortality [18,19]. The prominent clinical feature of cachexia is weight loss with anorexia, inflammation, insulin resistance, and increased muscle protein breakdown. Increased muscle protein breakdown in cachexia is at least partially due to accelerated muscle proteolysis by the ubiquitin-proteasome pathway, a common pathway of muscle mass loss due to pathological as well as physiological causes [20]. However, the signaling mechanism of the activation of the ubiquitin-proteasome pathway in cachexia appears to be different from that of physiological muscle atrophy. It is well established that a depression in AKT activity activates FoxO1/3 transcription factors, which upregulates two key ubiquitin ligases (E3 proteins), atrogin1/MAFbx and MuRF1, in animal models of physiological muscle atrophy caused by fasting, denervation and disuse [21-23]. In animal models of cachexia, however, AKT is often activated, which leads to the inactivation of FoxO1/3 [4,5,24]. In fact, it has been shown in animal models of cachexia that upregulation of MuRF1 is mediated by NF-κB [25], and upregulation of atrogin1/MAFbx is mediated by p38 MAPK [4,5].

We showed most recently that among the known p38 MAPK isoforms only p38β MAPK is capable of upregulating atrogin1/MAFbx via the activation of transcription factor C/EBPβ in response to tumor cell-conditioned medium. In addition, we demonstrated that p38β MAPK upregulation of atrogin1/MAFbx is independent of the AKT-FoxO1/3 signaling pathway [17]. Thus, p38β emerged as a key mediator and a specific therapeutic target of cachexia. Notwithstanding, why p38β is uniquely capable of activating C/EBPβ among the p38 isoforms is unknown. The current study is designed to address the mechanism through which p38β specifically activates C/EBPβ in the context of tumor-induced cachexia. We demonstrate that while p38β shares some common phosphorylation sites with p38α, it specifically phosphorylates the Thr-188 residue of C/EBPβ, which activates C/EBPβ binding to the atrogin1/MAFbx promoter and upregulates this gene in response to a tumor.

## Methods

Tryptic phosphopeptide mapping was carried out to identify p38α- and p38β-mediated phosphorylation sites in C/EBPβ. ChIP assay was used to evaluate p38α and p38β effect on C/EBPβ-binding to the atrogin1/MAFbx promoter. Overexpression or siRNA-mediated gene knockdown of p38α and p38β, and site-directed mutagenesis or knockout of C/EBPβ, were used to analyze the roles of these kinases in muscle catabolism in C2C12 myotubes and mice.

### Tryptic phosphopeptide mapping

HEK293T cells (American Type Culture Collection, Manassas, VA, USA) cultured in 150 mm culture plates that were ~50% confluent were co-transfected with a plasmid encoding LAP with a FLAG tag (Addgene) and a plasmid encoding constitutively active p38α or p38β [26] (10 μg each) using deacylated polyethylenimine (PEI) 22000 [27], a gift from Dr. Guangwei Du (University of Texas Health Science Center at Houston, Houston, TX, USA). The cell culture medium was replaced with fresh medium at 24 h. Cells were lysed in RIPA buffer (50 mM Tris–HCl (pH 7.5), 150 mM NaCl, 2 mM EDTA, 1% NP-40, 0.1% SDS, 2 mM phenylmethylsulphonylfluoride (PMSF), 0.5% sodium deoxycholate, 1 mM NaF, 1/100 protease inhibitor cocktail, and 1/100 phosphatase inhibitor cocktail (Sigma-Aldrich, St. Louis, MO, USA) after an additional incubation of 24 h. LAP in cell lysates was precipitated using FLAG-M2 magnetic beads (Sigma-Aldrich) and subjected to 10% SDS-PAGE. The gel was then stained with Coommassie Blue R-250. The LAP band was cut out and subjected to tryptic phosphopeptide mapping conducted by Taplin Mass Spectrometry Facility at Harvard Medical School using an LTQ-Orbitrap mass spectrometer (Thermo Electron, West Palm Beach, FL, USA).

### Myogenic cell culture and transfection

Murine C2C12 myoblasts (American Type Culture Collection, Manassas, VA, USA) were cultured in growth medium (DMEM supplemented with 10% fetal bovine serum) at 37°C under 5% CO_2_. At approximately 85 to 90% confluence, myoblast differentiation was induced by incubation for 96 h in differentiation medium (DMEM supplemented with 4% heat-inactivated horse serum) to form myotubes. Plasmids encoding constitutively active p38 isoforms [26] or a C/EBPβ mutant (p3xFlag-CMV-10-LAP-T188A) were transfected into C2C12 myoblasts of 50% confluence at 1 μg/well in six-well plates using deacylated polyethylenimine (PEI) 22000. At 24 h we induced the cells to differentiate by switching them to differentiation medium. When indicated, myotubes were treated with Lewis lung carcinoma cell (National Cancer Institute, Bethesda, MD, USA)-conditioned medium (LCM, 25% final volume) [17] or directly harvested. Cell lysate was prepared using the RIPA buffer for further analyses.

### Chromosome immunoprecipitation assay

Chromosome immunoprecipitation (ChIP) assay was performed as previously described [17].

### Generation of expression vector for a C/EBPβ mutant (LAP-T188A)

A plasmid encoding C/EBPβ in which Thr-188 was replaced by alanine in the pcDNA3 vector (a gift from Dr. Qi-Qun Tang of Fudan University, Shanghai, China) was digested at BamHI and EcoRI restriction sites to release the cDNA insert. That insert was then subcloned into the p3xFlag-CMV-10 vector (Sigma-Aldrich) at the same restriction sites to generate plasmid p3xFlag-CMV-10-LAP-T188A.

### Western blot analysis

Cell and muscle lysate were prepared and western blot analysis was carried out as described previously [17]. Antibodies for total and/or phosphorylated ATF2, FoxO1 (Thr-24)/FoxO3a (Thr-32) and C/EBPβ phosphorylated at Thr-188 were from Cell Signaling Technology (Danvers, MA, USA). Antibody for atrogin1/MAFbx was from ECM Biosciences (Versailles, KY, USA). Antibodies to C/EBPβ (H-7) were from Santa Cruz Biotechnology (Santa Cruz, CA, USA). Antibody to the HA tag was from Covance (Princeton, NJ, USA). Data were normalized to GAPDH.

### Gene knockdown by siRNA

p38α-specific siRNA (5′-CUCCUUUACUAUCUUUCUCAA-3′) and p38β-specific siRNA (5′-GUCCUGAGGUUCUAGCAAAdTdT-3′) were synthesized by Sigma-Aldrich and transfected into C2C12 myoblasts by electroporation (5 μg/1 × 10^7^ cells) with the Nucleofector system (Lonza, Walkersville, MD, USA), according to the manufacturer’s protocol. Control siRNA was obtained from Ambion (Austin, TX, USA). Differentiation was induced 24 h after transfection.

### Animal use

Experimental protocols were approved in advance by the institutional Animal Welfare Committee at the University of Texas Health Science Center at Houston. C/EBPβ^−/−^ mice in C57BL/6 background were bred from C/EBPβ^−/+^ mice generated by Dr. Peter Johnson of NCI [28]. While the mice were under anesthesia, plasmids encoding constitutively active p38α or p38β were injected into the tibialis anterior (TA) of the right leg for each mouse (100 μg in 50 μl), and the empty vector pcDNA3.1 was injected into the left leg as control. Immediately after plasmid injection, TA was electroporated by applying square-wave electrical pulses (100 V/cm) eight times with an electrical pulse generator (Model 830, BTX) at a rate of one pulse per second, with each pulse being 20 ms in duration, through a pair of stainless steel needles that were 5 mm apart. The above transfection procedure was repeated in 7 days. In another 7 days, the mice were sacrificed and TAs were collected for analysis.

### Statistical analysis

Data were analyzed with one-way ANOVA or student *t* test using the SigmaStat software (Systat Software, Point Richmond, CA, USA) as indicated. When applicable, control samples from independent experiments were normalized to a value of 1 without showing variations (actual variations were within a normal range). A *P* value <0.05 was considered to be statistically significant. Data are presented as the mean ± S.E.

## Results

### p38β MAPK specifically phosphorylates the Thr-188 residue of C/EBPβ

**Figure 1:**
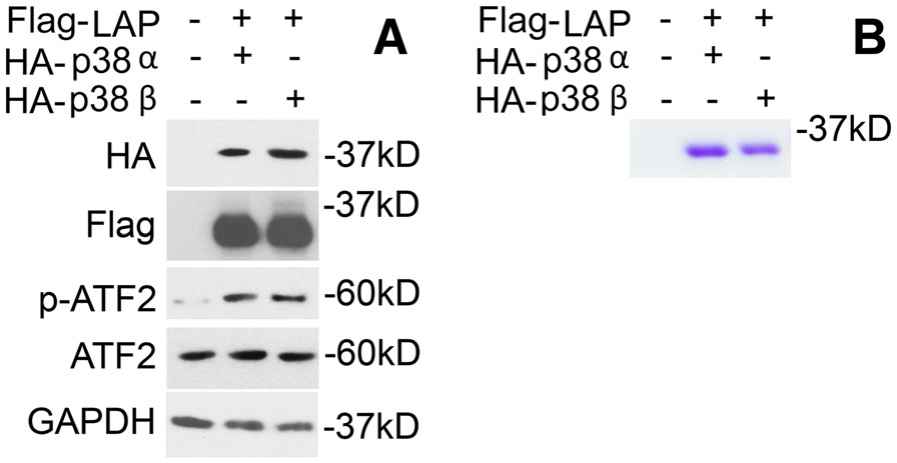
**Isolation of C/EBPβ phosphorylated by p38α or p38β MAPK for tryptic phosphopeptide mapping.** HEK293T cells were co-transfected with plasmids encoding C/EBPβ (LAP fused with the FLAG tag) and constitutively active p38α or p38β (fused with the HA tag). After 48 h incubation the cells were lysed. Expression of the transfected plasmids was verified by western blot analysis of HA and FLAG. Activity of over-expressed p38α and p38β was evaluated by western blot analysis of ATF2 activation (**A**). Overexpressed C/EBPβ was pulled down with FLAG-M2 magnetic beads and separated by SDS-PAGE. The gel was stained with Coommassie Blue R-250 (**B**). The C/EBPβ band was then cut out and analyzed by tryptic phosphopeptide mapping utilizing mass spectrometry for phosphorylated amino acid residues. MAPK, mitogen-activated protein kinase.

C/EBPβ is a transcription factor that is normally repressed due to the intrinsic repression of its DNA-binding and transactivation functions [29,30]. The DNA-binding function of C/EBPβ is activated by sequential phosphorylation of specific amino acid residues by multiple kinases [31-33]. C/EBPβ was previously shown to be a p38 substrate *in vitro*[34]. Recently, we showed that p38 interacts with and phosphorylates C/EBPβ in C2C12 myotubes. In addition, while the p38α/β inhibitor SB202190 blocks tumor-induced atrogin1/MAFbx upregulation, only p38β is capable of upregulating atrogin1/MAFbx via the activation of C/EBPβ [17]. To investigate why p38β, but not p38α, has the capacity for activating C/EBPβ we set out to investigate the phosphorylation sites in C/EBPβ that are targeted by p38α and p38β. Plasmids encoding C/EBPβ (LAP fused with the FLAG tag) and constitutively active p38α or p38β (fused to HA) were co-transfected into HEK293T cells. At 48 h expression of the transfected plasmids was verified by western blot analysis of HA (p38 MAKPs) and FLAG (LAP) in the cell lysate. Activation of p38 substrate ATF2 (via phosphorylation) by expressed p38 MAPKs was also evaluated by western blot analysis (Figure 1A). Overexpressed C/EBPβ was pulled down with FLAG-M2 magnetic beads and separated with SDS-PAGE (Figure 1B). The C/EBPβ band was cut out from the gel and analyzed by tryptic phosphopeptide mapping utilizing mass spectrometry (for original reports see Additional file 1: Table S1 and Additional file 2: Table S2). Six phosphorylated amino acid residues were identified in C/EBPβ that was co-expressed with active p38α and eight phosphorylated amino acid residues were identified in C/EBPβ that was co-expressed with active p38β. The two p38 MAPK isoforms shared four common phosphorylation sites (Ser-64, Ser-184, Ser-222, and Ser-276). On the other hand, p38α uniquely phosphorylated Ser-110 and Tyr-108, while p38β uniquely phosphorylated Ser-182, Ser-183, Ser-190 and Thr-188 (Table 1). Among the unique amino acid residues phosphorylated by p38β, Thr-188 is known to be crucial for the activation of C/EBPβ binding to its targeted DNA sequence [31-33].

**Table 1:** **Expression of constitutively active p38**α **or p38β resulted in the phosphorylation of diverse amino acid residues in C/EBPβ**

**Kinase**	**Phosphorylated amino acid residues in C/EBPβ**
p38α	Ser110, Tyr108
p38β	Ser182, Ser183, Ser190, Thr188
p38α/p38β	Ser64, Ser184, Ser222, Ser276

The C/EBPβ bands cut out from SDS-PAGE described in Figure 1B were analyzed by tryptic phosphopeptide mapping utilizing mass spectrometry. Identified phosphorylated amino acid residues in C/EBPβ are listed.

**Figure 2:**
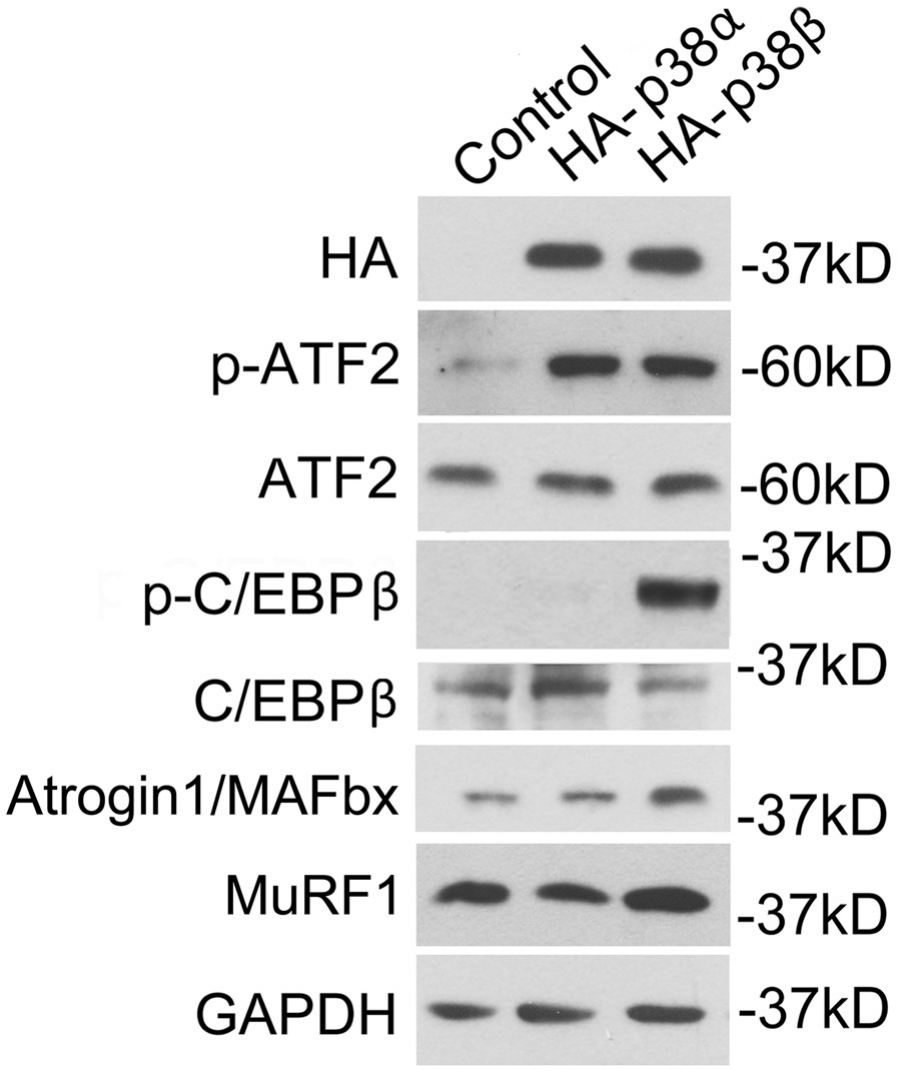
**Expression of constitutively active p38β in C2C12 myotubes resulted in specific phosphorylation of C/EBPβ at Thr-188 and upregulation of atrogin1/MAFbx.** C2C12 myoblasts were transfected with a plasmid encoding constitutively active p38α, p38β or the empty vector (control). The myoblasts were allowed to differentiate for 96 h to form myotubes that were then harvested and analyzed for ATF2 activation, Thr-188 phosphorylation in C/EBPβ and level of atrogin1/MAFbx using western blotting.

To verify whether p38β specifically mediates the phosphorylation of Thr-188 of C/EBPβ in muscle cells, we transfected C2C12 myoblasts with plasmids encoding active p38α or active p38β and used empty vector as the control. Although constitutively active p38α appeared to accelerate differentiation during the early stage of differentiation, at 96 h of differentiation, there was no visible difference in the myotubes formed compared with control myotubes and active p38β-expressing myotubes. After differentiation, lysate of myotubes was evaluated by western blot analysis of expression of the HA tag that fused to active p38α or p38β, ATF2 activation and C/EBPβ phosphorylation at Thr-188. As shown in Figure 2, although both of the active p38 isoforms activated ATF2, only the expression of active p38β resulted in C/EBPβ phosphorylation at Thr-188 and upregulation of atrogin1/MAFbx. Therefore, the specific phosphorylation of Thr-188 by p38β may be the key to its specific activation of C/EBPβ.

### p38β MAPK-mediated Thr-188 phosphorylation activates C/EBPβ binding to the atrogin1/MAFbx promoter and upregulates atrogin1/MAFbx in C2C12 myotubes

**Figure 3:**
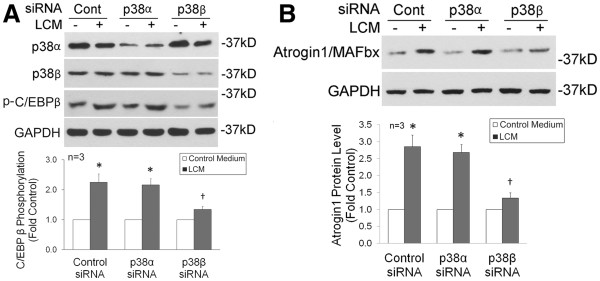
**p38β is critical to LCM-induced phosphorylation of C/EBPβ at Thr-188 and upregulation of atrogin1/MAFbx.** C2C12 myoblasts were transfected with siRNA as indicated. After differentiation, myotubes were treated with LCM or control medium. In 1 h, levels of p38α and p38β, and phosphorylation of C/EBPβ at Thr-188 were evaluated by western blotting (**A**). In 8 h, levels of atrogin1/MAFbx were evaluated by western blotting (**B**). Optical density of the bands that represent C/EBP phosphorylated at Thr-188 or atrogin1/MAFbx was analyzed by ANOVA. *denotes a difference from control without LCM treatment and †denotes a difference from control with LCM treatment (*P* <0.05). LCM, Lewis lung carcinoma cell-conditioned medium.

To verify whether p38β is critical to Thr-188 phosphorylation and atrogin1/MAFbx upregulation induced by cachectic tumor cells, siRNA-mediated mRNA knockdown was carried out. We observed that knockdown of the p38β expression, but not the p38α expression, blocked Lewis lung carcinoma cell-conditioned medium (LCM)-induced Thr-188 phosphorylation (Figure 3A) and atrogin1/MAFbx upregulation (Figure 3B). Therefore, p38β is indeed a key mediator of Thr-188 phosphorylation and atrogin1/MAFbx upregulation by Lewis lung carcinoma.

**Figure 4:**
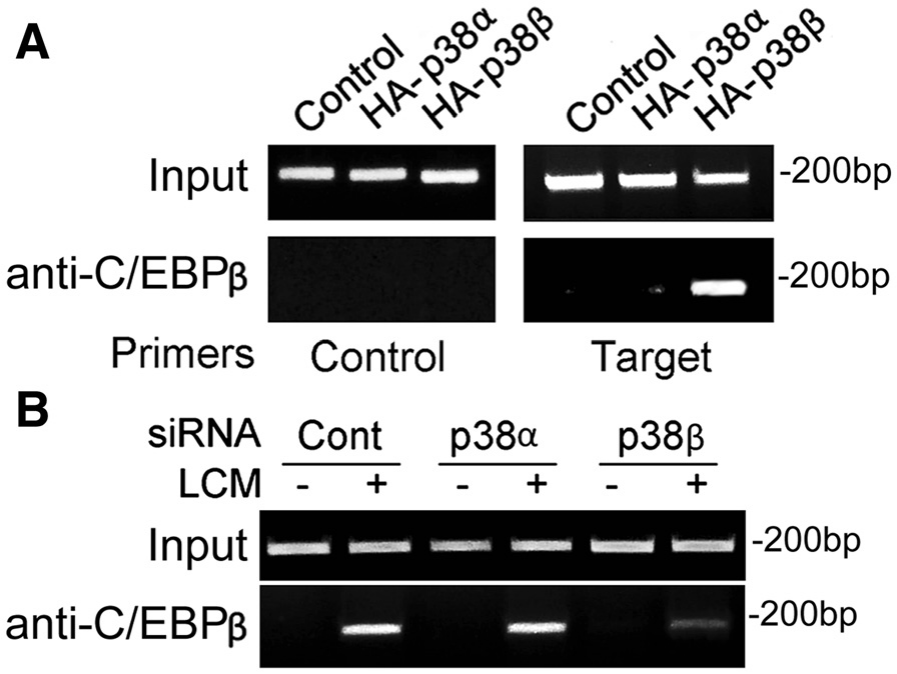
**p38β specifically activates C/EBPβ-binding to the atrogin1/MAFbx promoter.** (**A**) C2C12 myoblasts were transfected with a plasmid encoding constitutively active p38α, p38β or the empty vector (control). After differentiation, ChIP assay was carried out to evaluate C/EBPβ binding in myotubes to a 190-base pair fragment of the atrogin1/MAFbx promoter that contains the previously identified C/EBPβ-responsive cis-enhancer element [17] using control and the target PCR primers. Pre-immune IgG used as the control to the antibody against C/EBPβ did not pull down the target fragment (data not shown). (**B**) C2C12 myoblasts were transfected with siRNA as indicated. After differentiation, myotubes were treated with LCM or control medium for 1 h and ChIP assay was carried out to evaluate C/EBPβ binding to the atrogin1/MAFbx promoter. ChIP, chromosome immunoprecipitation; LCM, Lewis lung carcinoma cell-conditioned medium.

Previously, we observed that MAPK kinase 6 (MKK6) activates C/EBPβ binding to the C/EBPβ-responsive enhancer in the atrogin1/MAFbx promoter via the activation of p38 [17]. To investigate whether p38β specifically activates C/EBPβ binding to this C/EBPβ-responsive enhancer, we conducted the ChIP assay. We observed in C2C12 myotubes that the expression of active p38β, but not active p38α, activated C/EBPβ binding to the atrogin1/MAFbx promoter region containing the C/EBPβ-responsive enhancer (Figure 4A). Conversely, siRNA-mediated knockdown of the p38β gene, but not the p38α gene, blocked LCM-induced activation of C/EBPβ binding to the atrogin1/MAFbx promoter (Figure 4B). Thus, we conclude that p38β specifically activates C/EBPβ binding to its targeted DNA motif.

**Figure 5:**
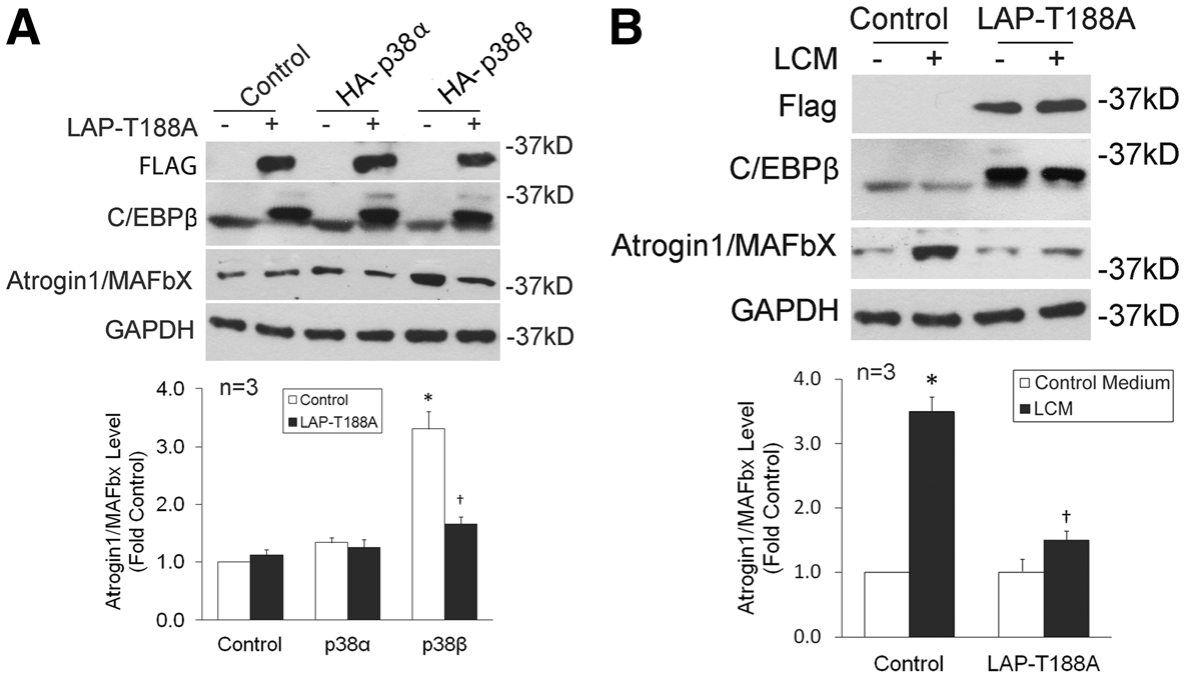
**C/EBPβ phosphorylation at Thr-188 is crucial for atrogin1/MAFbx upregulation by p38β or LCM.** (**A**) C2C12 myoblasts were transfected with a plasmid encoding constitutively active p38α, p38β or empty vector. A plasmid encoding FLAG-tagged C/EBPβ mutant in which Thr-188 was replaced with alanine (LAP-T188A) or the control vector was co-transfected as indicated. After differentiation, myotubes were lysed and analyzed by western blotting for the expression of LAP-T188A (with antibodies against FLAG and C/EBPβ) and atrogin1/MAFbx. (**B**) C2C12 myoblasts were transfected with LAP-T188A or the empty vector as control. After differentiation, myotubes were treated with LCM or control medium for 8 h. Lysate of myotubes was analyzed by western blotting for the expression of LAP-T188A (with antibodies against FLAG and C/EBPβ) and atrogin1/MAFbx. Optical density of the bands that represent atrogin1/MAFbx was analyzed by ANOVA. *denotes a difference from control without LCM treatment and †denotes a difference from control with LCM treatment (*P* <0.05). LCM, Lewis lung carcinoma cell-conditioned medium.

To evaluate whether C/EBPβ phosphorylation at Thr-188 is critical to p38β-mediated upregulation of atrogin1/MAFbx, a plasmid encoding a C/EBPβ mutant in which Thr-188 was replaced by alanine (C/EBPβ-T188A) was transfected into C2C12 myoblasts along with a plasmid encoding active p38α or active p38β. After differentiation, levels of atrogin1/MAFbx in myotubes that expressed active p38β, but not active p38α, were elevated. The elevation of atrogin1/MAFbx was blocked in myotubes co-expressing the C/EBPβ-T188A mutant (Figure 5A). To evaluate whether tumor-induced atrogin1/MAFbx upregulation requires the phosphorylation of C/EBPβ at Thr-188, the plasmid encoding C/EBPβ-T188A was transfected into C2C12 myoblasts. After differentiation, myotubes were treated with LLC cell-conditioned medium (LCM). We observed that LCM upregulation of atrogin1/MAFbx was inhibited in myotubes that overexpress the C/EBPβ-T188A mutant (Figure 5B). Therefore, p38β-mediated C/EBPβ phosphorylation at Thr-188 is crucial for atrogin1/MAFbx upregulation by LLC cells.

### p38β MAPK specifically induces atrogin1/MAFbx upregulation and muscle mass loss in mice via C/EBPβ

**Figure 6:**
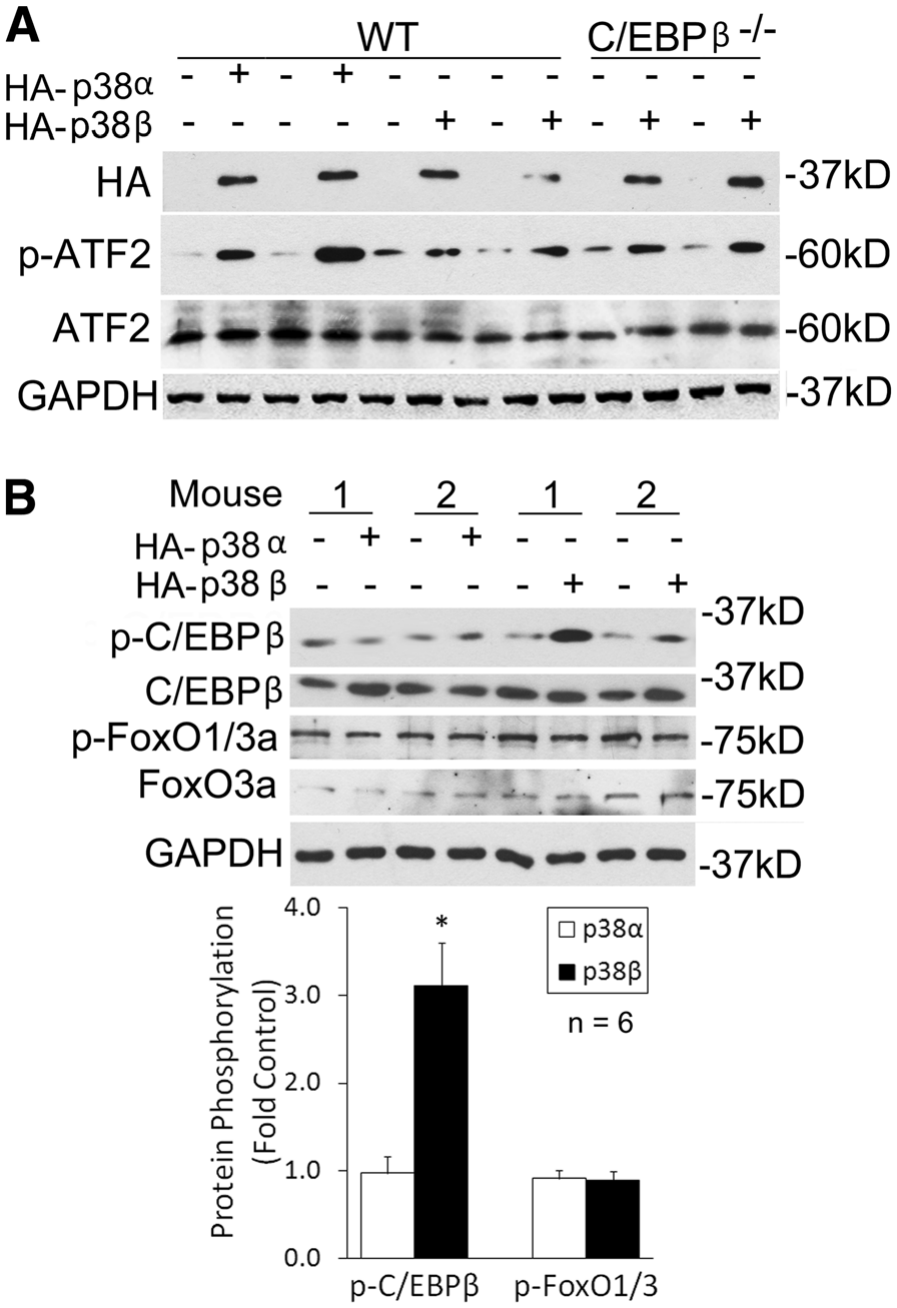
**Expression of constitutively active p38β in mouse muscle induces C/EBPβ phosphorylation at Thr-188.** Plasmids encoding constitutively active p38α or p38β were transfected into TA of the right leg of wild type or C/EBPβ^−/−^ mice, and the empty vector into the left leg. In 14 days TA samples were collected, weighed and lysed. Expression of the p38 isoforms was verified by western blot analysis of HA tag expression and ATF2 activation. A representative blot is shown (**A**). The TA lysate was subjected to western blot analysis of C/EBPβ phosphorylation at Thr-188 and FoxO1/3 activation. Representative blots and densitometry data are shown. Optical density of the bands that represent various proteins was measured. Levels of protein phosphorylation were normalized to that of total proteins. *denotes a difference (*P* <0.05) based on Student *t* test (**B**). TA, tibialis anterior.

**Figure 7:**
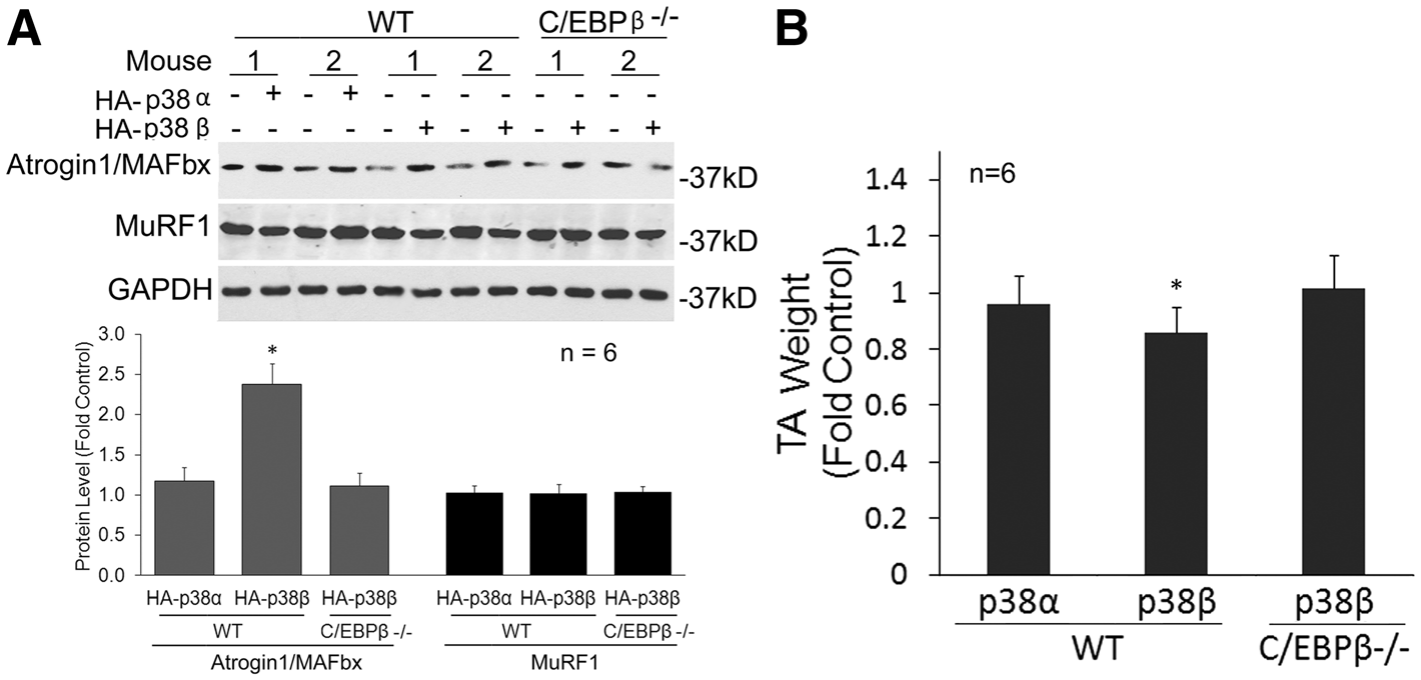
**Expression of constitutively active p38β in mouse muscle induces atrogin1/MAFbx upregulation and muscle mass loss in a C/EBPβ-dependent manner.** The TA lysate derived from Figure 6 was further analyzed for level of atrogin1/MAFbx by western blotting. Representative blots and densitometry data are shown (**A**). The weight of TA transfected with a plasmid encoding an active p38 isoform was compared to that of TA transfected with empty vector (control) from the same mouse (**B**). *denotes a difference (*P* <0.05) based on ANOVA. TA, tibialis anterior.

We previously showed that C/EBPβ is essential for the atrogin1/MAFbx upregulation and muscle mass loss in Lewis lung carcinoma (LLC)-tumor bearing mice [17]. To evaluate whether activation of p38β *in vivo* specifically induces C/EBPβ phosphorylation at Thr-188, atrogin1/MAFbx upregulation and muscle mass loss, the plasmids encoding constitutively active p38α or p38β were transfected into the tibialis anterior (TA) of wild type or C/EBPβ^−/−^ mice with the empty vector as control. At 14 days, the mice were sacrificed and the excised TAs were analyzed. Expression of the HA tag that fused to p38α or p38β and activation of the p38 substrate ATF2 were evaluated by western blot analysis to confirm the expression of active p38α and p38β (Figure 6A). Expression of active p38β, but not active p38α, resulted in phosphorylation of C/EBPβ at Thr-188 (Figure 6B). Because the atrogin1/MAFbx promoter also contains FoxO1/3-responsive cis-elements that are regulated by AKT [21], we evaluated whether p38β affected FoxO1/3 activity. Expression of active p38β did not alter the phosphorylation state of FoxO1/3 (Figure 6B). Therefore, p38β does not affect FoxO1/3 activity. As shown in Figure 7A, expression of active p38β resulted in atrogin1/MAFbx upregulation in the TA of wild type mice. However, active p38β failed to upregulate atrogin1/MAFbx in C/EBPβ^−/−^ mice. Finally, expression of active p38β, but not active p38α, induced TA weight loss by 14% in wild type mice. In contrast, active p38β expression did not alter TA weight in C/EBPβ^−/−^ mice (Figure 7B). Therefore, *in vivo* activation of p38β induces atrogin1/MAFbx upregulation and muscle mass loss via the activation of C/EBPβ.

## Discussion

The selective activation of substrates by various p38 MAPK isoforms was previously attributed to preferential activation of the isoforms by specific MAPK kinase as well as compartmentalization of the isoforms. For example, while p38α is activated by MKK3, MKK6 and MKK4, p38β is activated by MKK6 [10,35]. However, these may not explain the specific activation of C/EBPβ by p38β, because both p38α and p38β are present in the nucleus and activated by MKK6. The current study demonstrates that the selective activation of substrates by p38 MAPK isoforms is further realized by their recognition of specific phosphorylation sites within a substrate.

Particularly, we show that p38β specifically mediates the phosphorylation of C/EBPβ required for the activation of its binding to the atrogin1/MAFbx promoter. Previous studies indicated that phosphorylation of Thr-188 in C/EBPβ by ERK1/2 MAPK [31] or cdk2/cyclinA [32] primes C/EBPβ for subsequent phosphorylation on Ser-184 or Thr-179 by glycogen synthase kinase 3β (GSK3β), which activates the DNA-binding and transactivation functions of C/EBPβ [33]. Our data presented here demonstrate that p38β has the unique capability of mediating dual phosphorylation at both Thr-188 and Ser-184, resulting in the activation of C/EBPβ binding to the atrogin1/MAFbx promoter. In contrast, p38α that mediates phosphorylation of Ser-184 but not Thr-188 is unable to activate C/EBPβ binding to the atrogin1/MAFbx promoter. Because GSK3β was previously shown inactivated by p38 MAPK-mediated phosphorylation [36], it is unlikely that GSK3β mediates C/EBPβ phosphorylation at Ser-184 in response to p38α and p38β activation.

In the present study we also present the first evidence that overexpression of active p38β in muscle induces muscle catabolism, demonstrating a direct effect of p38β on muscle catabolism *in vivo*. Previous studies involving systemic activation of p38 in tumor-bearing mice [17] or septic mice [4] did not allow such a conclusion.

Although p38β is widely distributed in various tissues [37] its function is largely unknown, especially when it is compared to p38α, which is not only responsible for the known roles of p38 in inflammatory responses [38,39] but also in the regulation of myogenesis [12,13]. The present study demonstrates that activation of p38β, not p38α, induces atrogin1/MAFbx upregulation and muscle mass loss via specific phosphorylation of C/EBPβ, which explains at the molecular level why p38 is capable of playing the seemingly opposing roles in muscle protein homeostasis (promoting myogenesis *versus* promoting muscle catabolism). Further, these data support p38β as a selective therapeutic target of cachexia. Because C/EBPβ is activated by a number of kinases and regulates a wide variety of genes [40], it may not be suitable as a drug target. On the other hand, p38β has few known functions, therefore, specific inhibitors of p38β MAPK would be highly desirable for the intervention of cancer cachexia. Unfortunately, only p38α/β-dual or p38α-specific inhibitors are available at the present time.

Because p38β is highly expressed in the heart [37], it may also regulate the protein homeostasis in heart muscle via influencing C/EBPβ−regulated atrogin1/MAFbx expression. Consistent to this notion, it has been shown recently that exercise induces a reduction in C/EBPβ in cardiomyocytes, which mediates cardiomyocyte hypertrophy [41]. In addition, the transactivation activity of C/EBPβ is suppressed by insulin, an anabolic hormone [42]. Therefore, diverse signaling pathways that regulate protein homeostasis in striated muscles may converge upon C/EBPβ.

## Conclusions

The present study demonstrates that the α and β isoforms of p38 MAPK recognize distinct phosphorylation sites in a substrate. p38β MAPK has the unique capacity to mediate the dual phosphorylation of Thr-188 and Ser-184 in C/EBPβ, thereby, activating this transcription factor and inducing muscle catabolism. Therefore, p38β MAPK should be considered as a therapeutic target for cachexia.

## Abbreviations

ChIP: Chromosome immunoprecipitation; COPD: Chronic obstructive pulmonary disease; DMEM: Dulbecco’s modified Eagle’s medium; LCM: Lewis lung carcinoma cell-conditioned medium; LLC: Lewis lung carcinoma; MAPK: Mitogen-activated protein kinase; MKK6: MAPK kinase 6; NF-κB: Nuclear factor-κB; PCR: Polymerase chain reaction; siRNA: Small interfering RNA; TA: Tibialis anterior; TNF-α: tumor necrosis factor-α.

## Competing interests

The authors declare that they have no competing interests.

## Authors’ contributions

GZ performed the experiments, analyzed the data, and generated the figures. Y-PL designed the research and wrote the article. Both authors read and approved the final manuscript.

## Supplementary Material

Additional file 1 C/EBPβ Phosphorylation Sites By p38α. Click here for file

Additional file 2 C/EBPβ Phosphorylation Sites By p38β. Click here for file
