# Supplementary figures and images for: p38β MAPK upregulates atrogin1/MAFbx by specific phosphorylation of C/EBPβ
Source: Skelet Muscle. 2012 Oct 9;2:20. doi: 10.1186/2044-5040-2-20 (PMC3534568; doi:10.1186/2044-5040-2-20)

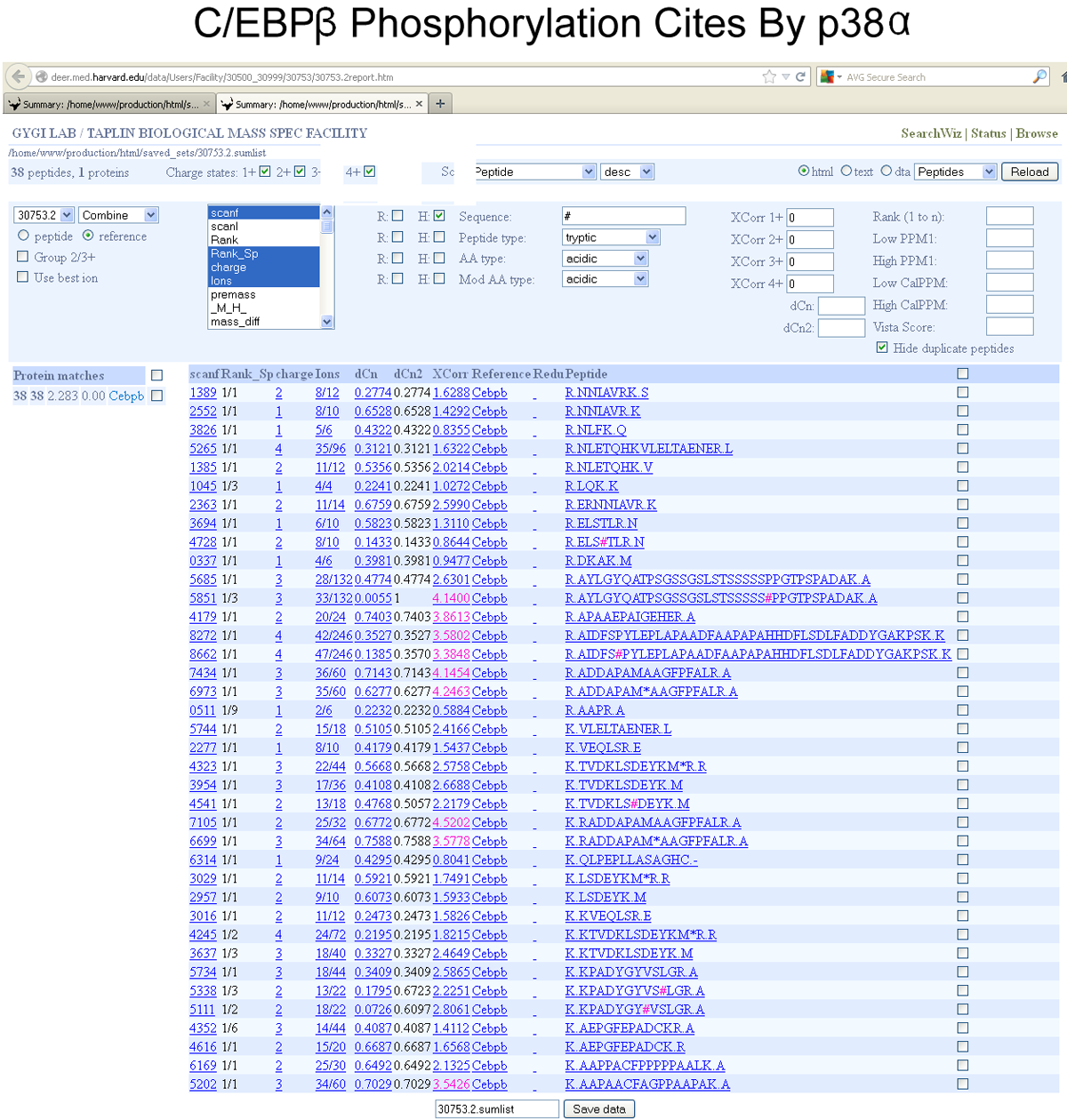

Supplement: Additional file 1 — C/EBPβ Phosphorylation Sites By p38α. [file 2044-5040-2-20-S1.tiff]

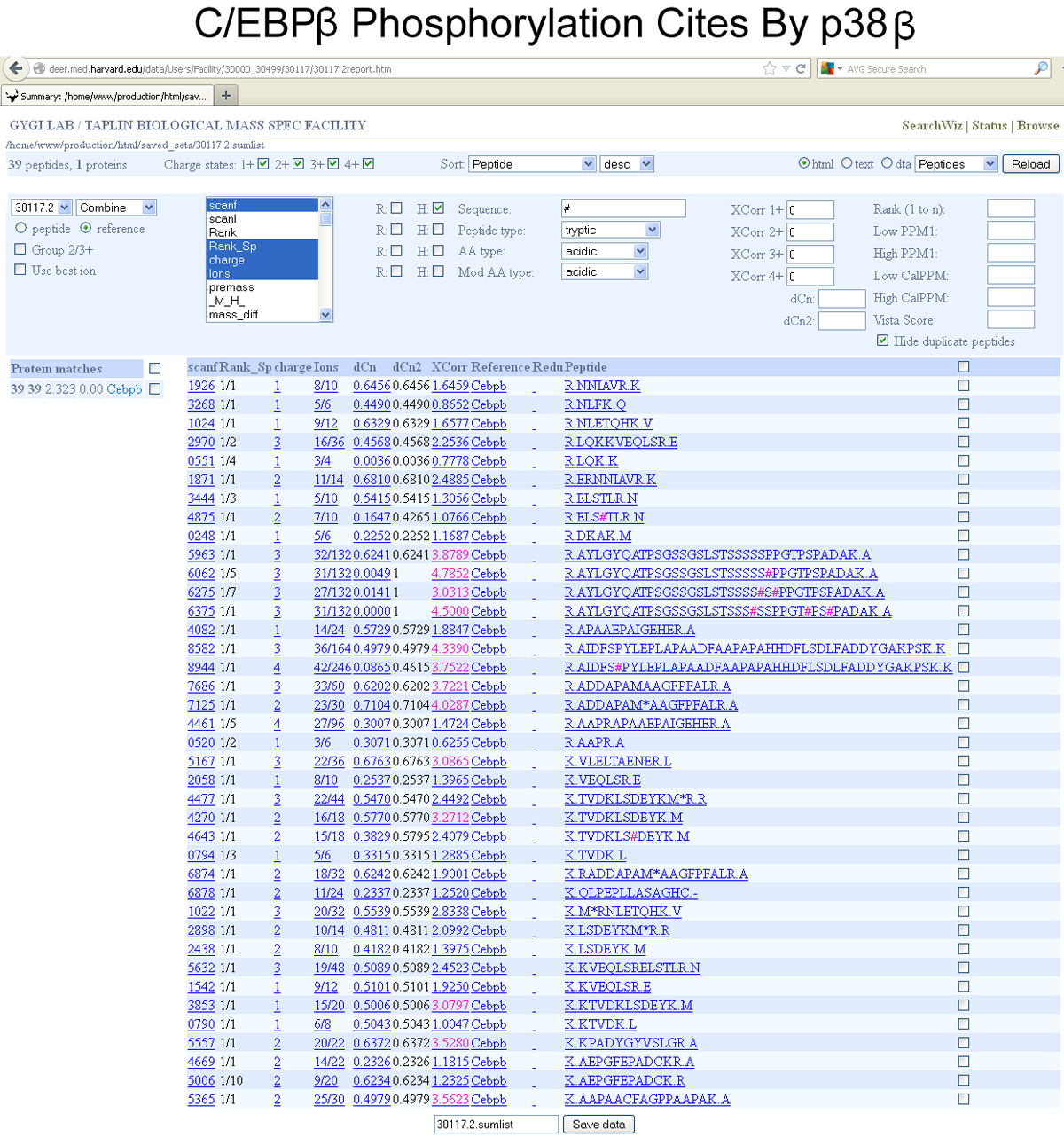

Supplement: Additional file 2 — C/EBPβ Phosphorylation Sites By p38β. [file 2044-5040-2-20-S2.tiff]
